# ‘Palliative-D’—Vitamin D Supplementation to Palliative Cancer Patients: A Double Blind, Randomized Placebo-Controlled Multicenter Trial

**DOI:** 10.3390/cancers13153707

**Published:** 2021-07-23

**Authors:** Maria Helde Frankling, Caritha Klasson, Carina Sandberg, Marie Nordström, Anna Warnqvist, Jenny Bergqvist, Peter Bergman, Linda Björkhem-Bergman

**Affiliations:** 1 Department of Neurobiology, Care Sciences and Society (NVS), Division of Clinical Geriatrics, Karolinska Institutet, SE-141 83 Huddinge, Sweden; maria.helde.frankling@ki.se (M.H.F.); caritha.klasson@ki.se (C.K.); 2 ASIH Stockholm Södra, Palliative Home Care and Hospice Ward, SE-125 59 Älvsjö, Sweden; 3 Stockholms Sjukhem, Palliative Medicine, SE-112 19 Stockholm, Sweden; Carina.a.sandberg@gmail.com (C.S.); marie.k.nordstrom@gmail.com (M.N.); 4 Department of Environmental Medicine, Division of Biostatistics, Karolinska Institutet, SE-171 77 Stockholm, Sweden; anna.warnqvist@ki.se; 5 Department of Surgery, Breast Centre, Capio St Gorans Hospital, SE-112 19 Stockholm, Sweden; jenny.bergqvist@capiostgoran.se; 6 Department of Oncology-Pathology, Karolinska Institutet, SE-171 77 Stockholm, Sweden; 7 Department of Laboratory Medicine, Division of Clinical Microbiology, Karolinska Institutet, SE-141 86 Stockholm, Sweden; peter.bergman@ki.se; 8 Department of Infectious Diseases, Immunodeficiency Unit, Karolinska University Hospital, SE-141 86 Stockholm, Sweden

**Keywords:** vitamin D, Detremin, cholecalciferol, supplementation, randomized clinical trial, placebo, palliative, cancer, fatigue, quality of life, antibiotics, ESAS, EORTC QLQ-C15-PAL

## Abstract

**Simple Summary:**

In this study, the effect of vitamin D supplementation on pain, infections, fatigue and quality of life in patients with advanced cancer with verified vitamin D deficiency was studied. To this end, a randomized controlled trial, ‘Palliative-D’, was conducted, comparing the effect of 4000 IU vitamin D_3_/day for 12 weeks to placebo in cancer patients admitted to palliative care. Pain was assessed as change in opioid dose and infections measured as days on antibiotics. Vitamin D-supplemented patients increased their opioid doses at a significantly slower rate than patients receiving placebo, i.e., 0.56 µg less fentanyl/h per week with vitamin D treatment. Vitamin D reduced self-assessed fatigue but did not affect antibiotic use or self-assessed Quality of life. The treatment was safe and well-tolerated. In conclusion, correction of vitamin D deficiency may have positive effects on pain and fatigue in palliative cancer patients.

**Abstract:**

The aim of the ‘Palliative-D’ study was to test the hypothesis that correction of vitamin D deficiency reduces opioid use in cancer patients admitted to palliative care. A multicenter randomized, placebo-controlled, double-blind trial in three home-based palliative care facilities in Sweden was performed. Patients with advanced cancer and 25-hydroxyvitamin D < 50 nmol/L were randomized to vitamin D3 4000 IU/day or placebo for 12 weeks. The primary endpoint was the difference of long-acting opioid use (fentanyl ug/h) between the groups during 12 weeks, based on four time points. Secondary outcomes included changes in antibiotic use, fatigue and Quality of Life (QoL). A total of 244 patients were randomized, and 150 patients completed the 12 weeks. The major reason for drop-out was death due to cancer. The vitamin D-group had a significantly smaller increase of opioid doses compared to the placebo-group; beta coefficient −0.56 (*p* = 0.03), i.e., 0.56 µg less fentanyl/h per week with vitamin D treatment. Vitamin D-reduced fatigue assessed with ESAS was −1.1 points after 12 weeks (*p* < 0.01). Antibiotic use or QoL did not differ significantly between the groups. The treatment was safe and well-tolerated. In conclusion, correction of vitamin D deficiency may have positive effects on opioid use and fatigue in palliative cancer patients, but only in those with a survival time more than 12 weeks.

## 1. Introduction

Vitamin D is a steroid hormone that maintains calcium homeostasis and skeletal health [1]. Vitamin D has also been investigated for its effects on the immune and nervous systems [2,3]. 25-hydroxyvitamin D (25-OHD) is used to assess an individual’s vitamin D status in routine clinical settings [1]. Levels of 25-OHD lower than 50 nmol/L are considered insufficient, and below 20 nmol/L as severely insufficient [4]. Vitamin D induces the synthesis of antimicrobial peptides on mucosal surfaces, on the skin and in immune cells, supporting the immune response to infections [5]. Vitamin D also reduces cytokine release, dampens inflammatory T-cell responses and has been shown to downregulate prostaglandin synthesis [6,7,8,9]. These findings provide a possible mechanistic explanation for effects of vitamin D supplementation regarding infections and pain.

Previous randomized controlled trials have shown that vitamin D supplementation reduced antibiotic use and respiratory tract infections [10,11,12].

Vitamin D supplementation has been studied in clinical cohorts experiencing different types of pain, including musculoskeletal pain, migraine as well as visceral and neuropathic pain, with both positive and negative results [2,13,14]. A recent study showed that patients with vitamin D deficiency (25-OHD < 25 nmol/L) who had undergone surgery were at increased risk for higher opioid use compared to those with normal vitamin D levels [15]. Previous trials have shown that vitamin D supplementation is beneficial only in patients with low levels of 25-OHD at baseline [13,16]. Furthermore, patients diagnosed with cancer have lower 25-OHD levels than healthy controls from the same latitude [17,18,19,20,21]. Interestingly, vitamin D supplementation has been associated with decreased cancer mortality [22].

A cross-sectional observational study at our palliative care facility revealed that lower levels of 25-OHD were associated with prescription of higher doses of opioids [17]. Based on this observation, we performed a pilot study, where 39 patients with vitamin D deficiency were supplemented with vitamin D_3_ oil drops (4000 IU/day) for 12 weeks [23]. Compared to matched, untreated patients from the observational cohort, these patients received lower opioid doses after one month, and had fewer days on antibiotic treatment after three months. QoL assessed with Edmonton Symptom Assessment Scale (ESAS) [24] improved significantly in the intervention group [23]. To test whether these results could be reproduced in a randomized and placebo-controlled setting, we designed the ‘Palliative-D’ study.

## 2. Materials and Methods

### 2.1. Study Design

‘Palliative-D’ was a multicenter, double-blind parallel group, 1:1, randomized, placebo-controlled trial performed at three palliative care facilities in Stockholm, Sweden. Detailed information on the clinical units is provided in Supplementary Files. During the trial period, four changes were made to the original design (Supplementary Files).

### 2.2. Ethical Statement

The study protocol was published before the start of the trial [25] and registered at Clinicaltrial.gov: NCT03038516. The study was approved by the Regional Ethical Committee in Stockholm (Dnr 2017/405-31/1) and was conducted according to the declaration of Helsinki. Written informed consent was obtained from all participants before any study related procedure was performed.

### 2.3. Participants

Included patients were admitted to one of the three recruiting palliative care facilities, ≥18 years old, had advanced and/or metastatic cancer in palliative phase (any type of cancer), a life expectancy of at least three months as assessed by one of the three study physician and 25-OHD ≤ 50 nmol/L. Ongoing oncological treatment was allowed, but not with intention to cure. Exclusion criteria at screening were 25-OHD > 50 nmol/L, hypercalcemia during the past two months; eGFR < 30 mL/h; a medical history of kidney stones, sarcoidosis and/or primary hyperparathyroidism; current medication including vitamin D > 400 IU/day, digoxin/digitoxin or thiazides; hypersensitivity to the study drug; participation in other clinical trials involving medication; or other reasons for not being able to complete the planned procedures. Ongoing opioid treatment at screening was not required for participation in the trial. Written informed consent was obtained from all participants before any study-related procedures were performed. Full inclusion and exclusion criteria are given in the study protocol [25].

### 2.4. Randomization and Masking

Patients were randomly assigned 1:1 to vitamin D_3_ oil drops (color and taste matched) 4000 IU/day or placebo (oil drops) for 12 weeks, dispensed in identical, sequentially numbered bottles. Randomization was performed using a computer-generated randomization list to generate a permutated block randomization with a block size of four. Randomization was not stratified. Trial masking for patients, trial staff and care providers continued until data had been analyzed. Detremin 20,000 IU/mL (cholecalciferol solved in Miglyol oil) and placebo (Miglyol oil) were prepared according to Good Manufacturing Practice by Nextpharma. Eurofins LC2, a centralized randomization unit, was responsible for labeling, blinding and randomization. Detremin was provided from Renapharma, Uppsala, Sweden, and placebo was from Nextpharma, Limay, France.

### 2.5. Interventions

After written informed consent was collected, patients’ records were reviewed. Information on age, sex, antibiotic treatment during the past 30 days, current dose of long-acting opioid at the day of screening, ongoing oncological treatment and type of cancer was retrieved. Blood samples were collected for analysis of 25-OHD (methods in Supplementary Files), CRP, albumin, creatinine and calcium, and for biobanking. Patients completed the Edmonton Symptom Assessment Scale (ESAS) [24] and EORTC QLQ-C15-PAL [26], described further in Supplementary Files.

Patients with 25-OHD ≤ 50 nmol/L, no hypercalcemia and eGFR > 30 were eligible for randomization. We informed all patients about their baseline 25-OHD-level. Data on opioid dose and antibiotic use were collected once more at baseline.

Study visits were every fourth week (+/−7 days) in connection with regular weekly visits in patients’ homes (Figure S1). Each study visit included a blood sample for analysis of albumin, calcium, creatinine and CRP, completion of ESAS and collection of data on current opioid dose and antibiotic use during the past month. At the end of study (visit 3; 12 weeks), patients were once more asked to complete EORTC QLQ-C15-PAL, and additional blood-samples were taken for measurement of 25-OHD (blinded for the study team) and for storage in a biobank. Assessment of compliance is outlined in the Supplementary Files, as are the procedures after the end of the study.

### 2.6. Outcomes

The predefined primary outcome was the mean difference in change in long-acting opioid dose between the treatment arms, measured as fentanyl ug/hour during 12 weeks, based on four time points: 0, 4, 8 and 12 weeks, and adjusted for baseline opioid-values. In a secondary analysis, adjustments were made also for age, sex, oncological treatment, baseline 25-OHD and colectomy. The null hypothesis was that the fentanyl dose would increase with same rate in both groups, whereas the alternative hypothesis was that the fentanyl dose would increase faster in one of the groups. The choice of fentanyl dose as a primary outcome was based on the positive results in the pilot study [23] and is further described in the Supplementary Files.

Secondary outcomes were mean difference in change on antibiotic use, fatigue and QoL, assessed with ESAS and EORTC QLQ-C15-PAL, and 25-OHD-levels between the treatment arms after 12 weeks. Antibiotic use, as a proxy for infections, was measured as the number of days with antibiotics in the previous 30 days. Fatigue was assessed with the “tiredness” question in the ESAS form and with Question 11 in EORTC QLQ-C15-PAL. QoL was assessed with the QoL-question in ESAS, and Question 15 in EORTC QLQ-C15-PAL.

Patients were withdrawn from the study if they developed hypercalcemia (albumin-adjusted calcium > 2.60), if eGFR dropped below 30 mL/min, if they were prescribed medications that were not allowed according to the study protocol, had poor compliance, could no longer take the study drug, withdrew consent, were lost to follow-up or reported serious or intolerable adverse events.

### 2.7. Monitoring of Adverse Events

Detremin has few and mild side effects, mostly nausea and diarrhea. Intoxication of vitamin D may lead to hypercalcemia or in worst case renal failure, but this is extremely rare. Daily doses of 4000 IU/day have not resulted in toxic 25-OHD concentrations in previous studies [10,12,27]. According to the study protocol, only GI-symptoms, an increase in creatinine levels, hypercalcemia and renal failure needed to be recorded as adverse events.

### 2.8. Sample Size

When calculating the sample size, we used results from relevant distributions in the pilot study [23], and considered 20% to be a clinically relevant effect size. We aimed for a targeting power of 80%, with a significance level of 0.05 (two sided) regarding the primary outcome. The sample size of 190 patients resulted in an estimated power of 81.6%, and with an expected dropout rate of 25%, the estimated sample size was concluded to be 254 [25]. Detailed information on sample size calculation is presented in Supplementary Files.

### 2.9. Statistical Analyses

Variables were summarized using median and interquartile range (Table S1). Primary analysis was performed using linear mixed effect regression using information from three time points: 4, 8 and 12 weeks, adjusting for the baseline value. The baseline values were categorized (Table S2). Linear mixed models with person specific random intercepts and slopes were used to control for the intra-person correlation inherent to repeated measurements. The parameter of interest was the interaction of group and time, interpreted as the average difference in weekly opioid dose change between groups. As secondary analysis, adjustments were made for baseline opioid use, age, sex, oncological treatment, colectomy and baseline 25-OHD levels (Table S3). Both intention-to-treat (ITT) and per-protocol (PP) analyses were performed for the primary outcome in accordance with the study protocol.

The difference in opioid doses at 12 weeks was analyzed using linear regression. As with the primary analysis, two models were analyzed; unadjusted and adjusted. Estimation of confidence intervals (CI) was performed using normal approximation (see Supplementary Files).

All secondary outcomes were analyzed at week 12 using only linear regression, as pre-specified in the protocol. Adjustments were made as for the primary outcome. Antibiotic treatment was categorized at baseline: 0, 1–7, 8–14 and 15–30 days.

Due to the high death rates during follow-up, several sensitivity analyses were performed as described by Carpenter et al. [28].

The number needed to treat (NNT) was calculated using the reduction of at least 12 units of fentanyl, from baseline to the end of study at 12 weeks, as indicating a positive treatment effect. The difference in the percentage attaining the reduction of 12 units in both groups was calculated and the inverse of the difference was taken to calculate the NNT.

To investigate mortality in the treatment groups, survival functions were estimated using the Kaplan–Meier estimator and log-rank test for equality.

All analyses were performed in Stata 15 (StataCorp. 2017. Stata Statistical Software: Release 15. College Station, TX: StataCorp LLC.). Multiple imputation was done using the STATA command mimix [29].

## 3. Results

**Figure 1:**
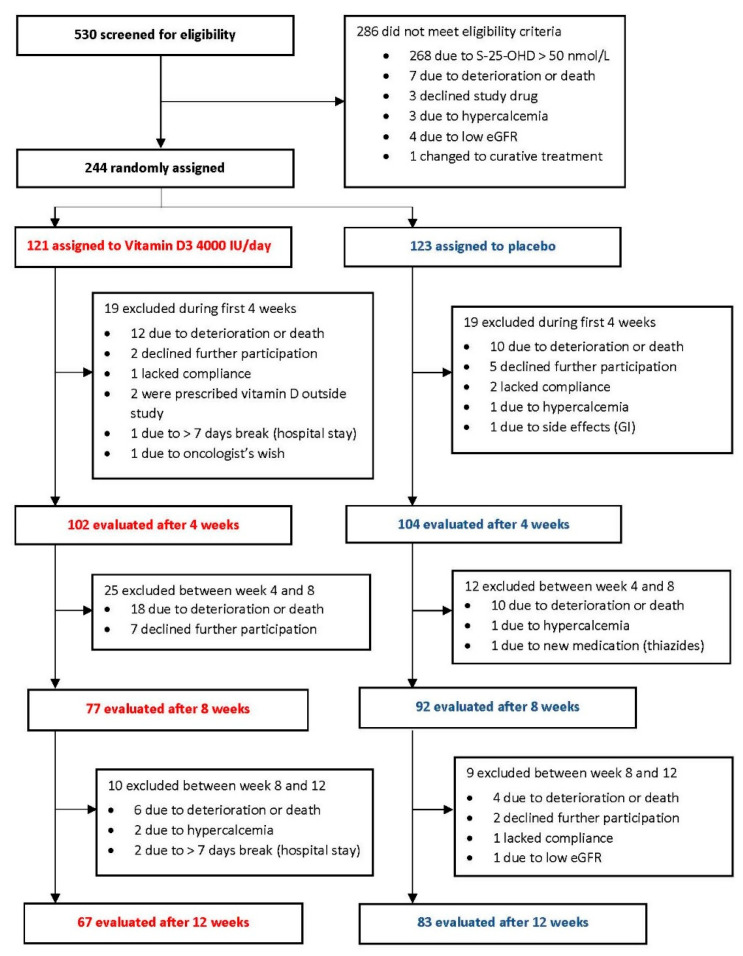
Flow chart of the included and excluded patients in the ‘Palliative-D’ study.

‘Palliative-D’ was conducted between November 2017 and June 2020. A total of 530 patients were screened, and 244 patients were randomized to receive the study drug (ITT population), 121 received vitamin D_3_ and 123 received a placebo (Figure 1). Inclusion stopped early due to the COVID-19 pandemic, and 10 patients fewer than planned were randomized. A total of 150 patients completed all 12 weeks of vitamin D (*n* = 67) or placebo (*n* = 83) and constitute the PP population (Figure 1). The major reason for drop-out was death due to cancer. Baseline demographics from the screened cohort (*n* = 530) have been published previously [30].

A total of 244 patients were included in the ITT analysis, which was based on 769 observations and four time points over 12 weeks. Groups were well balanced at baseline (ITT: Table 1 and PP: Table S1).

**Figure 2:**
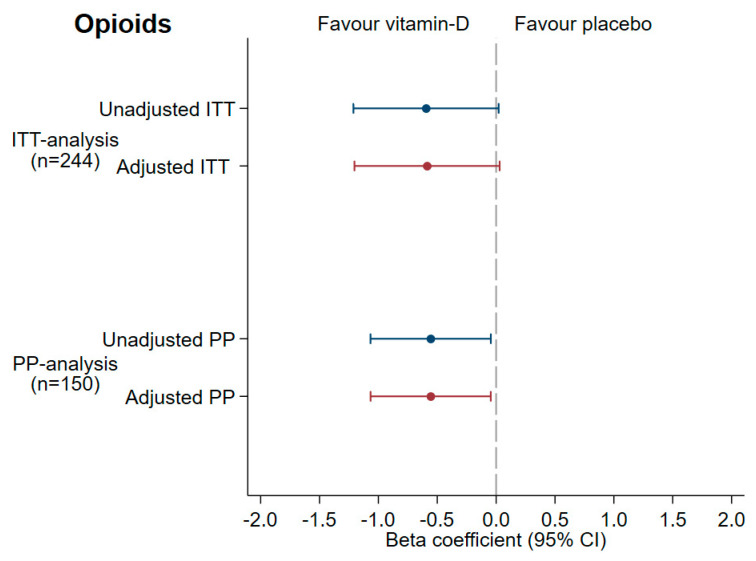
Main analysis: forest plots with beta coefficient and 95% CI over the opioid use in the ‘Palliative-D’ study. The intention-to-treat (ITT) analyses include all 244 randomized patients, and the Per Protocol (PP) analysis is based on the 150 patients that completed the 12-week study period (vitamin D 4000 IE/day *n* = 67 and placebo *n* = 83). Adjustments were made for the baseline in all analyses and for age, sex, oncological treatment, baseline 25-hydroxyvtaim D and colectomy in the “adjusted model”.

The ITT analysis did not show a significant difference between the slopes, beta coefficient −0.60 (95% CI −1.21; 0.02; *p* = 0.06), and in the adjusted analysis, beta −0.59 (95% CI −1.20 to 0.03; *p* = 0.06) (Figure 2). The sensitivity analyses of different imputation models are shown in Table S3.

However, in the PP analysis (*n* = 150), based on 450 observations, the mean increase in opioid doses in the vitamin D group was significantly smaller than in the placebo group, beta coefficient −0.56 (95% CI −1.07; −0.05; *p* = 0.03), in both the unadjusted and adjusted analysis, i.e., 0.56 µg less fentanyl/h and week with vitamin D treatment (Figure 2), corresponding to 6.72 ug/h after 12 weeks. Thus, the null hypothesis that there would be no difference between the slopes could be rejected in the PP analysis.

A separate non-longitudinal analysis performed on data after 12 weeks only also showed significantly lower opioid doses in the vitamin D arm, −7.0 µg /h (*p* = 0.03) (Table S4).

The number needed to treat (NNT) was calculated to be 12 patients for 12 weeks to decrease the opioid dose with at least 12 µg fentanyl/hour in one patient. Self-assessed pain did not differ between the two treatment arms during the study period, indicating that opioid doses were adequately adjusted (Supplementary Files).

**Figure 3:**
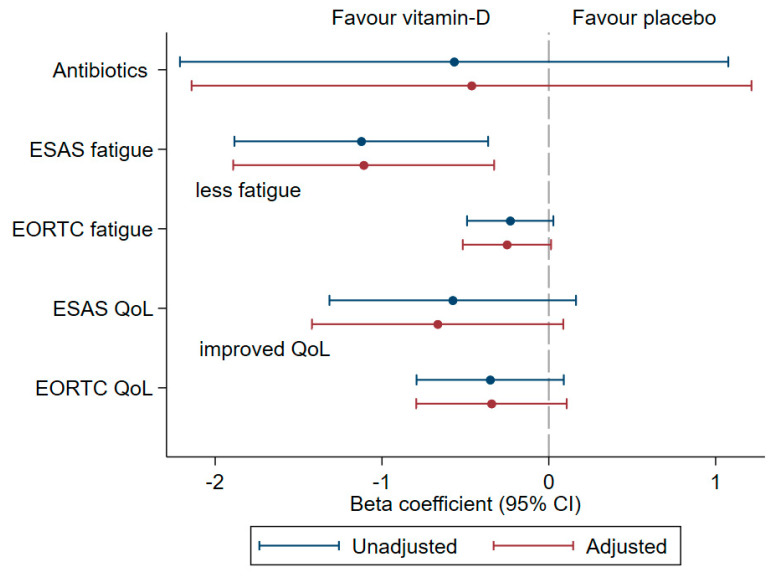
Secondary outcomes: forest plot with beta coefficient and 95% CI over the antibiotic consumption, fatigue and QoL assessed with ESAS and EORTC QLQ-C15-PAL in the ‘Palliative-D’ study. The analysis is based on the Per Protocol (PP) analysis of the 150 patients completing the 12-week study period (vitamin D 4000 IE/day *n* = 67 and placebo *n* = 83). Adjustments were made for the baseline in all analyses and for age, sex, oncological treatment, baseline 25-hydroxyitamin D and colectomy in the “adjusted model.” The scale of EORTC QLQ-C15-PAL has been reversed so the negative value of beta is an improvement in QoL, in line with all the other outcomes where negative value shows improvement.

After 12 weeks of treatment, there was no significant difference in antibiotic use or QoL between treatment arms (Figure 3).

The vitamin D group exhibited a significantly lower degree of fatigue assessed with ESAS compared to the placebo group after 12 weeks; −1.1 point (*p* < 0.01) (Figure 3). Fatigue assessed with EORTC QLQ-C15-PAL was not significantly different between the groups (*p* = 0.06) (Figure 3).

Vitamin D treatment increased mean 25-OHD significantly, from 36 (±11) nmol/L to 81 (±26) nmol/L (*p* < 0.001), while mean 25-OHD in the placebo group remained stable (Figure S2).

Overall survival was not a predefined endpoint in ‘Palliative-D,’ but we observed large drop-out rates that differed between treatment arms (Figure 1). Thus, we conducted a post-hoc survival analysis (Figure S3). Notably, there was no difference in survival time between the two treatment arms at any timepoint, after 4 weeks (*p* = 0.36), 8 weeks (*p* = 0.09) or 12 weeks (*p* = 0.08).

The unadjusted raw data at each time point in the ITT and the PP study populations for both primary and secondary outcomes are presented in the Supplementary Files. There was now a significant difference between the treatment arms regarding any outcome after 4 or 8 weeks (Figures S4 and S5).

There was no difference in calcium, creatinine, albumin or CRP levels between the two treatment arms throughout the study (Figures S6 and S7).

One patient developed renal failure during the study and the code was broken in this case. Unmasking of the treatment allocation revealed that this patient had received placebo. No other serious adverse events (SAE) were recorded. Treatment was well tolerated, with two cases of mild hypercalcemia in the vitamin D group and two in the placebo group (adverse events are shown in Table S5).

## 4. Discussion

Here, we show that correction of vitamin D deficiency is safe and may have positive effects on opioid use and fatigue. After 12 weeks, the mean opioid dose (fentanyl) in the vitamin D group was 6.7 µg/h lower than in the placebo group. NNT analysis revealed that 12 patients had to be treated for 12 weeks to obtain a reduction for one patient with the clinically relevant dose 12 µg/h (smallest available fentanyl patch).

Vitamin D-supplemented patients exhibited less fatigue compared to the placebo group, with a reduction of >1 ESAS point, a change that is assessed as clinically relevant [31,32]. However, vitamin D supplementation had no effect on antibiotic use. No difference in QoL between groups could be observed. As expected, vitamin D treatment significantly increased 25-OHD levels.

A strength of the ‘Palliative-D’ study is the study design. RCTs are difficult to conduct in palliative care [33,34,35,36,37], and thus, most data from palliative care facilities are based on observational or case-control studies [38]. Furthermore, data are comprehensive, with very few missing data points in patients completing the study. The mean increase in 25-OHD after 12 weeks of vitamin D treatment (42 nmol/L) was somewhat larger than in the pilot study (33 nmol/L), indicating good compliance. Since we included patients with all types of advanced cancer with varying remaining life span, generalizability of the results may be broad. Significant results regarding opioid dose and fatigue were obtained, although only 150 patients, 40 fewer than planned, completed all 12 weeks. Vitamin D treatment in this group proved safe and well tolerable, and screening for 25-OHD deficiency would be feasible considering analytical costs.

The major limitation of this study was the large drop-out rate, with fewer patients completing all 12 weeks in the intervention group, resulting in loss of power and increased risk of both Type I and type II errors. The large drop-out rate highlights the difficulties in performing clinical trials in late-stage cancer patients and for even trained palliative care professionals to estimate remaining life span in patients with advanced cancer, often overestimating survival time [39,40].

There was a larger drop-out rate in the vitamin D arm, especially between 4 and 8 weeks, although overall survival did not differ significantly between the two treatment arms at any time point. Still, it cannot be entirely ruled out that vitamin D in some way was detrimental, leading to higher drop-out rate in the vitamin D arm, although the mechanistic basis for such an effect remains unclear. However, it should be noted that vitamin D has not been shown to have a negative effect in other cancer-trials [41,42]. In the large VITAL study on 25,871 healthy subjects ≥ 50 years old randomized to vitamin D_3_ 2000 IE/day or placebo with a median follow-up time of 5.3 years, vitamin D actually reduced the incidence of metastatic or fatal cancer [41]. Overall, we believe that vitamin D is safe when given to cancer patients in the palliative phase, although careful monitoring for potential side-effects is recommended.

The observed effect size of −6.7 µg/h fentanyl per patient after 12 weeks might be of limited clinical importance. However, the results presented here underscore a new conceptual advancement that vitamin D may have direct mechanistic effects on the opioid system in humans. As such, vitamin D supplementation should be explored in cancer pain, since it may provide a safe and accessible way to reduce opioid use.

It should also be noted that the study population was predominantly Caucasian. Thus, the results might not be generalizable to more diverse patient populations.

The potential mechanism for a beneficial effect of vitamin D on opioid use remain elusive. Current evidence mainly supports effects of vitamin D on immune response, and thus, possibly inflammatory pain [6,8,9]. However, in a rat model for hyperalgesia, vitamin D was shown to reduce pain via induction of opioid-associated genes in the cerebrum and spinal cord [43]. Interestingly, a recent study showed that patients with vitamin D deficiency were more likely to have a diagnosis of an opioid use disorder [44]. In the same study, a direct mechanistic link between vitamin D signaling and opioid analgesia was provided by using several mouse models [44].

To the best of our knowledge, this is the first large RCT on vitamin D supplementation in advanced cancer patients measuring the effect of vitamin D on pain, infections, fatigue and QoL. Though there is continuous interest in the role of vitamin D in cancer prevention and treatment [22,45,46,47,48], data on the topic of vitamin D supplementation in symptom management of cancer patients are scarce and have often been collected from smaller cohorts and/or using a case-control design [18,23].

The results presented here suggest that vitamin D may improve fatigue, a symptom notoriously difficult to treat pharmacologically [49,50]. The findings are in accordance with our cross-sectional study of all screened patients at baseline in the ‘Palliative-D’ cohort (*n* = 530), showing a correlation between low 25-OHD and fatigue severity, especially in men [30]. The decreased need of opioids in the vitamin D group might contribute to decreased fatigue, as might decreased inflammatory immune response and prostaglandin synthesis [6,8,9]. However, a significant effect on fatigue was evident only when assessed with ESAS (*p* < 0.01) and not with EORTC QLQ-C15-PAL (*p* = 0.06). Thus, this finding should be interpreted with caution.

We did not observe effects of vitamin D supplementation on self-assessed QoL, in line with a previous study in patients with advanced cancer [51]. Although reduced pain and fatigue may contribute to better QoL, it may still not be enough to affect the overall life situation of these patients.

## 5. Conclusions

In conclusion, correction of vitamin D deficiency in cancer patients admitted to palliative care is safe, well-tolerated and may have a positive effect on opioid use and fatigue, but only in those with a survival time more than 12 weeks.

## Figures and Tables

**Table 1:** Baseline characteristics of randomized patients in the ‘Palliative-D’ study.

Variabel	All (*n* = 244)	Vitamin D (*n* = 121)	Placebo (*n* = 123)	*p*-Value
Age, median (IQR), years	68 (61–75)	68 (61–76)	68 (61–75)	0.64
Male, *n* (%)	120 (49)	62 (51)	58 (47)	0.61
Female, *n* (%)	124 (51)	59 (49)	65 (53)	0.61
No active oncological treatment, *n* (%)	78 (32)	37 (31)	41(33)	0.68
Chemotherapy, *n* (%)	118 (48)	58 (48)	60 (49)	0.90
Hormonal therapy, *n* (%)	28 (11)	18 (15)	10 (8)	0.11
Target therapy, *n* (%)	20 (8)	8 (7)	12 (10)	0.49
25-OHD, median (IQR), nmol/L	38 (28–45)	39 (28–45)	38 (28–45)	0.83
Fentanyl dose, median (IQR), ug/h	0 (0–25)	0 (0–37)	0 (0–25)	0.44
Prescribed long-acting opioid No. (%)	128 (52)	61 (50)	55 (45)	0.44
No. days on antibiotics, median (IQR), g/L	0 (0–3)	0 (0–1)	0 (0–3)	0.89
Albumin, median (IQR), g/L	30 (26–34)	30 (26–34)	30 (27–33)	0.88
Calcium, median (IQR), mmol/L	2.30 (2.21–2.38)	2.30 (2.19–2.36)	2.29 (2.23–2.38)	0.25
Creatinine, median (IQR), umol/L	70 (58–74)	68 (58–83)	71 (58–86)	0.54
CRP, median (IQR), mg/L	9 (3–31)	10 (3–27)	9 (3–31)	0.97
Colectomy No. (%)	31 (13)	17 (14)	14 (11)	0.57
ESAS fatigue, median (IQR)	4 (2–6)	4 (2–6)	4 (2–6)	0.88
ESAS QoL, median (IQR)	4 (2–6)	4 (2–6)	4 (2–6)	0.54
EORTC QLQ-C15-PAL, Q11 fatigue, median (IQR)	3 (2–3)	3 (2–3)	3 (2–3)	0.87
EORTC QLQ-C15-PAL Q15 QoL, median (IQR)	4 (3–5)	4 (3–5)	4 (3–5)	0.31
Type of cancer, No. patients				
Brain	2	1	1	>0.99
Breast	26	11	15	0.53
Upper gastrointestinal	59	30	29	0.88
Lower gastrointestinal	56	28	25	0.64
Gynecological	24	12	12	>0.99
Head and Neck	1	0	1	>0.99
Hematological	6	5	1	0.12
Lung	42	19	23	0.61
Melanoma	4	2	2	>0.99
Prostate	21	13	8	0.26
Sarcoma	3	1	3	0.62
Urinary tract	3	1	5	0.21

In the treatment group, two patients had two types of cancers (lung cancer + sarcoma and prostate cancer + upper GI cancer), and in the placebo, group two patients had two types of cancer (lung cancer + urinary tract cancer and upper GI cancer + prostate cancer). Systemic oncological treatment includes chemotherapy, targeted therapy, immunotherapy or a combination thereof. CI: Confidence interval, S-25-OHD: S-25-hydroxyvitamin D, ESAS: Edmonton Symptom Assessment Scale (range 0–10), EORTC QLQ-C15-PAL: European Organization for Research and Treatment of Cancer Quality of Life Questionnaire C15 Palliative (fatigue Q 11 range 1–4, QoL Q 15 range 1–7), QoL: Quality of Life Q: Question. A Mann–Whitney U test was used for continuous variables and Fisher’s exact test for categorical variables.

## Data Availability

Raw data are available from the corresponding author upon request.

## Supplementary Materials

The following are available online at https://www.mdpi.com/article/10.3390/cancers13153707/s1, Supplementary methods and results. Table S1: Baseline characteristics of patients who completed 12 weeks of intervention in the ‘Palliative-D’ study (= per protocol population), Table S2: Beta coefficients for the adjusted variables at baseline in the longitudinal model for opioid dose (primary outcome) in the ITT analysis, Table S3: Sensitivity analysis for the longitudinal linear mixed model, unadjusted ITT, Table S4: Effect of vitamin D 4000 IU/day after 12 weeks (non-longitudinal analysis) on opioid dose, antibiotic use, fatigue and quality of life (QoL) compared to placebo in the per-protocol analysis, Table S5: Adverse events in the ‘Palliative-D’ study in all randomized patients, Table S6: Opioid conversion table, Figure S1: Schematic Figure of data collection in the ‘Palliative-D’ study, Figure S2: 25-hydroxyvitamin D levels (25-OHD) in plasma at baseline and after 12 weeks of treatment, Figure S3: Kaplan–Meier plot of survival time, Figure S4: ITT population: raw data. i.e., not adjusted for baseline. Change in opioid doses, antibiotic use, fatigue and QoL, Figure S5: PP population: raw data. i.e., not adjusted for baseline. Change in opioid doses, antibiotic use, fatigue and QoL, Figure S6: ITT population: raw data. i.e., not adjusted for baseline. Levels of albumin-adjusted calcium, C-reactive protein (CRP), albumin and creatinine. Figure S7: PP population: raw data, i.e., not adjusted for baseline. Levels of albumin-adjusted calcium, C-reactive protein (CRP), albumin and creatinine.
