# Supplementary material for: ‘Palliative-D’—Vitamin D Supplementation to Palliative Cancer Patients: A Double Blind, Randomized Placebo-Controlled Multicenter Trial
Source: Cancers (Basel). 2021 Jul 23;13(15):3707. doi: 10.3390/cancers13153707 (PMC8345220; doi:10.3390/cancers13153707)
Supplement: Supplementary file 1 [file cancers-13-03707-s001.zip › cancers-1306694-supplementary.pdf]

cancers

MDPI

Supplementary Files

Amendments to the original study protocol

Amendments to the original protocol were obtained in Jan 2018 and Jan 2019: 1) From January 2018 the exclusion criteria were changed from exclusion of patients on any type of vitamin D treatment or supplementation to allowing recruitment of patients on daily doses of vitamin D up to 400 IE/day. The change was made since large number of patients met this exclusion criterion and since this dose was not considered to affect the endpoints. 2) We added eGFR <30 mL/h as an exclusion criterion for safety reasons. 3) In January 2019, a third site (ASIH Stockholm Norr) was added to increase inclusion rate. 4) The duration of the trial was extended from end of 2019 to June 2020 to enable the recruitment of all planned patients.

Description of the three palliative care facilities.

In Palliative D, patients were recruited from three different specialized home-based palliative care facilities in Stockholm, Sweden: ASIH Stockholm Södra, Stockholms Sjukhem and ASIH Stockholm Norr.

Swedish healthcare is government funded and decentralized. Responsibility for providing citizens with healthcare lies mostly with the 21 regional councils. The Stockholm Region has almost 2.4 million citizens, nearly a quarter of the population of Sweden.

In the Stockholm Region, specialized home-based palliative care facilities with multiprofessional teams admit patients with advanced or metastatic cancer who may or may not receive active oncological treatment. These teams can also admit curative cancer patients in need of a shorter period of home-based supportive care, as well as patients with non-cancer diseases in the palliative phase of their disease trajectory. Patients can choose any of the specialized home-based palliative care facilities authorized to provide care in their residential area (between two and nine different services, depending on area). These specialized home care facilities are all government-funded but can be owned and operated by the Stockholm Region Council as well as by private providers.

The average number of enrolled patients on any given day at ASIH Stockholm Södra is 380, at Stockholms sjukhem 240 and at ASIH Stockholm Norr 280. More than half of the enrolled patients are diagnosed with cancer. Patients are on average admitted for four months and include patients who receive advanced home care from a period of a few days to those who are admitted for periods longer than one year. All three facilities also operate palliative in-patients wards.

In 'Palliative-D', 328 patients were recruited from ASIH Stockholm Södra, 159 from Stockholms Sjukhem, and 44 from ASIH Stockholm Norr.

Patient enrolment

Study physicians screened all newly admitted patients to the participating facilities for cancer patients in the palliative phase of their disease trajectory. Study physicians then consulted with the patient's responsible physician before contacting patients (most often over the phone). Patients who expressed interest in participating in the study were sent written information and contacted again a few days later. If they were still interested in participating, an at-home screening visit was booked. Patients who fulfilled all inclusion and did not meet any exclusion criteria after the screening visit and after lab results returned, were randomized to study drug. A sequentially numbered box with two bottles of study drug was delivered to the patient within seven days (baseline visit).

The Edmonton Symptom Assessment Scale

ESAS is a psychometrically validated symptom assessment instrument that exists and is used in many different permutations [1]. It is used in clinical routine to quantify symptom burden in both cancer and non-cancer patient. ESAS is an eleven-point numeric

rating scale (NRS), rangning from 0 to 10, with ten different scale items. The symptoms include pain, dyspnea, loss of appetite, tiredness, drowsiness, nausea, wellbeing, anxiety and depressive symptoms. In addition, a 10th optional symptom can be added. In the Palliative-D study, the 10th optional symptom added is "quality of life." At zero, the verbal anchor is "no symptom", and at ten, the verbal anchor is "worst possible" symptom intensity. Patients are asked to assess symptoms at present. Symptom intensity scored as 1–3 as considered mild, 4–6 as moderate and 7–10 as severe. A change in one point on the 11-point scale is considered to be the minimal clinically important difference [2,3]. The version of ESAS used in study 'Palliative -D' is available in the study protocol. Fatigue was assessed with the "tiredness" question in ESAS.

EORTC-QLQ-C15-PAL

EORTC-QLQ-C15-PAL is a shortened version of the EORTC QLQ-C30, one of the most widely used QoL questionnaires in oncology, developed for palliative care patients. The form is comprised of 15 questions and assesses symptom / performance status during the past week [4]. Question number 11 is used to assess tiredness and is stated: "Were you tired?" The patient can score from 1–4, where 1="Not at all", 2="A little", 3="Quite a bit, 4="Very much". To assess fatigue with EORTC-QLQ-PAL15 it is sometimes suggested to use a combination of question number 11 and question number 7 using the instrument's scoring manual, generating results on a scale ranging from 0–100, where 0 is no fatigue and 100 is maximum fatigue. However, in this analysis we have decided to only use Q11 for fatigue assessment which is in accordance with our previous study on fatigue in this study cohort [5]. Quality of life is assessed by Q15 in the with EORTC-QLQ-PAL15 form: "How would you assess your total quality of life during the past week?" on a scale of 1–7, where the verbal anchor for 1 is "very poor QoL" and the verbal anchor for 7 is "Excellent QoL".

Sample size calculation

When calculating sample size, we used results from relevant distributions in the pilot study [6], and considered 20% to be a clinically relevant effect size. Based on the pilot study, the predicted mean dose for an untreated person after 12 weeks was 125 μg/h fentanyl, which, with an effect size of 20%, would translate to a difference between group means being -25μg/h. We aimed for a targeting power of 80%, with a significance level of 0.05 (two sided) regarding primary outcome. Since some of the distributions were skewed, 10.000 Monte Carlo simulations per sample size were performed. For each of the simulations, data were generated using the distributions of the previous study, and a linear regression model using bias corrected and accelerated (BCa) bootstrap was fit. The estimated power was the proportion of rejected null hypotheses. The sample size of 190 patients resulted in an estimated power of 81.6%, and with an expected dropout rate of 25%, the estimated sample size was concluded to be 254 [7].

Statistical analysis—calculation of CI

According to the original statistical analysis plan, the confidence intervals (CI) were to be estimated with the bias-corrected accelerated bootstrap method (Bca). However, a high proportion (20–35%) of the bootstrap replications were disregarded for several models. This was presumably due to lack of variation in the outcome, possibly causing a systematic bias. The attained results from the bootstrap-analyses were compared to the normal-approximation confidence intervals, and as the results were very similar and did not change the conclusions, the usual normal approximation CI's were used. In the comparisons, the normal approximation intervals were often wider than the Bca intervals.

Calculation of albumin adjusted calcium in plasma.

Albumin adjusted Calcium = Calcium + 0.01 × (39—Albumin).

Rationale for using fentanyl ug/hour as primary endpoint

Fentanyl dose was the main outcome in the pilot-study that had shown a positive result already after one month of vitamin D treatment [6]. Fentanyl patch is the most used opioid-treatment among the palliative care facilities in Stockholm and the dose is evaluated every week and adjusted accordingly. In patients treated with other opioids, a conversion table was used to assess fentanyl μg/hour (Table S6).

In our different specialized home-based palliative-care facilities patients receive weekly visits by a nurse. During this visit, patients are dispensed all their non-oncological medications for the coming week. Pain is assessed at every visit (biweekly using ESAS recorded in electronic medical records, every week through oral communication). Use of short acting opioids during the past week is assessed by inspection and oral communication. If use of short-acting opioids has increased or decreased during the past week, this reported back to the responsible physician, who can adjust the dose of long-acting opioid accordingly. If needed, an extra visit is scheduled so that patients need not to wait another week for change in long-acting opioid dose. Thus, patient's long-acting opioid doses are always up-to-date, and we therefore chose to use long-acting opioid dose assessed on a single day as primary endpoint. We chose to use fentanyl ug/hour, rather than morphine equivalent daily dose, since the majority of our patients are prescribed fentanyl patches. Fewer conversion calculations thus had to be made, minimizing bias from inaccurate conversion assessments.

In the adjusted model, adjustments were made for baseline opioid dose, age, sex and oncological treatment since we know from previous research and clinical experience that these factors may affect future change in opioid doses and pain [6,8,9,10]. We also know from our previous studies that patients with colectomy or short-bowel syndrome may have an impaired absorption of vitamin D from the gut [6,7,9,10], thus this variable was also adjusted for. Beta-values for these adjusting baseline variables are presented in Table S2.

Rationale for choices of secondary outcomes

Secondary outcomes included days of antibiotic use during the past month, as a proxy for bacterial infections. This was one of the outcomes in the pilot-study that had shown positive effects after 3 months of vitamin D treatment [6]. This is also the measure in previous studies on vitamin D treatment evaluating effect on infections [8,11,12]. Thus, this outcome was also chosen for the Palliative-D study.

Fatigue was assessed with the "tiredness" question in the ESAS which was the Question 11 outcome used in our previous study assessing fatigue in the baseline data of the Palliative-D cohort [5]. In concordance, when fatigue was measured with EORTC QLQ-C15-PAL only the tiredness question, Q11, was used for assessing fatigue and not the combined Q7 and Q11-score [5].

Method for measurements of 25-hydroxyvitamin D levels

Levels of 25-OHD in plasma were analyzed by chemiluminescence immunoassay (CLIA) on a LIAISON-instrument (DiaSorin Inc, Stillwater, MN, USA), detectable range 7.5–175 nmol/L, CV 2–5% at Dept of Clinical Chemistry, Karolinska University Hospital.

Procedures after end-of study

All patients were offered a bottle of Detremin after returning the study drug and were given the option to take 4000 IU/day. After 4 weeks of treatment, routine check-up of calcium, albumin and creatinine levels was performed by the study team. After these four weeks, responsibility for continued Detremin-use was transferred from the study team to the patient's responsible physician.

Compliance to study drug

We had three methods for assessing compliance:

Compliance was assessed by regular contact between the study nurse and participant after every follow-up visit during the intervention period.

Each patient was administered two bottles of study drug, and 1.5 bottles were used if patients took all planned doses during the study-period (12 weeks). At the final visit the participants were asked to return the study drug bottles for counting of bottles and oral inspection of the returned study drug.

In addition, 25-OHD levels at the end of study was also used as a measure of compliance (Figure S2).

Supplementary Results

Compliance in the vitamin D group

In the vitamin D group, 2 out of the 67 patients who completed the study reported failure in compliance during part of the study due to a hospital stay. Their 25-OHD levels at the end of the study were 42 and 58 nmol/L. All other study participants reported sufficient compliance. Nevertheless, 6 of these participants had 25-OHD <50 nmol/L at the end of study. A total of 45 bottles from the 67 that were distributed in the vitamin D group were returned to the study team, and 44 of the bottles showed that the participants had consumed 75–100% of the dose. One participant had consumed 50% of the dose. In 22 cases no bottles were returned. The mean 25-OHD level in the vitamin D group was 81 nmol/L; range 34–166 nmol/L (Figure S2).

Compliance in the placebo group

In the placebo group all participants reported sufficient compliance. Bottles from 57 of 83 participants were returned to the study team and 55 had consumed 75–100% of the dose. Two participants had consumed 50% of the dose. In 26 cases no bottles were returned. The mean 25-OHD in the placebo group at end of study was 39 nmol/L, range.

Subgroup analysis of the opioid users

At baseline, 50% of patients in the Vitamin D group and 55% of patients in the placebo group were prescribed long-acting opioids. For those taking opioids at baseline, the placebo group had a lower median, 25 μg/h (IQR 12–75), than the Vitamin D group, 37 μg/h (IQR 12–50). However, the placebo group had a higher mean (54.6) than the Vitamin D group (44.5), indicating that this group had higher outliers. After twelve weeks, the opioid doses of those who were taking opioids at baseline had diverged between the two groups. The median opioid dose increased in the placebo group (median 37 μg/h, mean 58.5), but decreased in the Vitamin D group (median 31 μg/h, mean 40.1).

Survival and drop-out rate

Survival was not a predefined endpoint in 'Palliative-D', and a Kaplan-Meier analysis was not included in the analysis plan. Since we had large drop-out rates that differed between treatment arms we conducted a post-hoc survival analysis (Figure S3). There was no difference in survival time between the two treatment arms at any timepoint, after 4 weeks ($p = 0.36$), 8 weeks ($p = 0.09$) or 12 weeks ($p = 0.08$). Statistical analysis was performed by using log-rank test for equality of survivor functions. However, there was a statistically significant higher drop-out rate in the vitamin D arm between 4 and 8 weeks compared to the placebo-arm ($p = 0.02$). This was not due to more deaths in the vitamin D group; but instead, a higher rate of patients that declined further participation after 4 weeks compared to the placebo arm (Figure 1). Still, the higher drop-out rate in the vitamin D arm, especially during the two first months, is concerning. The median survival time for all randomized patients (n = 244) was 6.1 months (95% CI: 5.2 – 7.1), whereas the median survival time for those completing the study (n = 150) was 8.5 months (95% CI: 7.5 – 9.9).

There were more patients who did not complete all 12 weeks in the vitamin D group (45%) than in the placebo group (33%). In both groups, baseline opioid dose was higher for patients who were excluded, compared to those who could be evaluated after 12 weeks. The baseline median opioid dose for excluded patients was 12 µg/h in both groups, but the placebo group had a wider interquartile range (IQR 0–50) than the vitamin D group (IQR 0–37). This would indicate that excluded patients from the placebo arm had higher opioid use compared to excluded patients from the Vitamin D group. This is also reflected in the mean baseline values of opioid use for excluded patients, which were higher in the placebo group (36.1) compared to the Vitamin D group (27.9).

Of those who were excluded and were taking opioids at baseline, the placebo group had higher opioid doses (median 50 µg/h (IQR 12 -75), mean 62.4) than the Vitamin D group (median 25 µg/h (IQR 18.5–50, mean: 47.1).

This would suggest that the results of the primary analysis were not due to the greater proportion of lost to follow-up in the treatment group, as there is no indication that persons who were lost to follow-up in that group had higher initial values.

Pain measured by ESAS

The self-assessed pain by ESAS did not differ between the two treatment arms during the study period indicating that the opioid-dose was adequately adjusted when the patient experienced more pain. The mean ESAS-score for pain in the vitamin D group was 1.8 units (SD 2.2) at baseline and 1.9 (SD 2.3) after 12 weeks. The corresponding mean scores in the placebo group were 1.9 (SD 2.2) and 1.9 (SD 1.9), respectively.

Outcome at each time in ITT- and PP-study populations

A separate analysis was performed in the ITT study population ($n = 244$) at each timepoint, i.e. after 4, 8 and 12 weeks in all randomized patients (Figure S4). This analysis showed that there was no difference between the treatment arms for opiod use, antibiotic use or QoL at any time point but for fatigue after 12 weeks.

The same analysis was performed in the PP-study population ($n = 150$) also showing that the difference between the treatment arms for opioid use and fatigue was evident first after 12 weeks (Figure S5). There was a significant difference for opioid use and fatigue after 12 weeks but not for antibiotic use or QoL at any time point.

Blood chemistry parameters

There was no difference in calcium (albumin adjusted) between the treatment arms in the ITT-study population at any time-point (Figure S6), nor for creatinine, albumin or CRP (Figure S6). Similarly, in the analysis including the patients completing 12 weeks, i.e. the PP-population, there was no difference between the treatment arms in any blood chemistry parameters over the study period (Figure S7).

References

1. Watanabe, S.M.; Nekolaichuk, C.L.; Beaumont, C. The Edmonton Symptom Assessment System, a proposed tool for distress screening in cancer patients: Development and refinement. Psycho. Oncol. **2012**, *21*, 977–985, doi:10.1002/pon.1996.
2. Bedard, G.; Zeng, L.; Zhang, L.; Lauzon, N.; Holden, L.; Tsao, M.; Danjoux, C.; Barnes, E.; Sahgal, A.; Poon, M.; et al. Minimal clinically important differences in the Edmonton symptom assessment system in patients with advanced cancer. J. Pain Symptom Manag. **2013**, *46*, 192–200, doi:10.1016/j.jpainsymman.2012.07.022.
3. Hui, D.; Shamieh, O.; Paiva, C.E.; Perez-Cruz, P.E.; Kwon, J.H.; Muckaden, M.A.; Park, M.; Yennu, S.; Kang, J.H.; Bruera, E. Minimal clinically important differences in the Edmonton Symptom Assessment Scale in cancer patients: A prospective, multicenter study. Cancer **2015**, *121*, 3027–3035, doi:10.1002/cncr.29437.
4. Groenvold, M.; Petersen, M.A.; Aaronson, N.K.; Arraras, J.I.; Blazeby, J.M.; Bottomley, A.; Fayers, P.M.; de Graeff, A.; Hammerlid, E.; Kaasa, S.; et al. The development of the EORTC QLQ-C15-PAL: A shortened questionnaire for cancer patients in palliative care. Eur. J. Cancer **2006**, *42*, 55–64, doi:10.1016/j.ejca.2005.06.022.
5. Klasson, C.; Helde-Frankling, M.; Sandberg, C.; Nordström, M.; Lundh-Hagelin, C.; Björkhem-Bergman, L. Vitamin D and Fatigue in Palliative Cancer: A Cross-Sectional Study of Sex Difference in Baseline Data from the Palliative D Cohort. J. Palliat. Med. **2021**, *24*, 433–437, doi:10.1089/jpm.2020.0283.

6. Helde-Frankling, M.; Höijer, J.; Bergqvist, J.; Björkhem-Bergman, L. Vitamin D supplementation to palliative cancer patients shows positive effects on pain and infections-Results from a matched case-control study. PLoS ONE 2017, 12, e0184208, doi:10.1371/journal.pone.0184208.
7. Helde-Frankling, M.; Bergqvist, J.; Klasson, C.; Nordstrom, M.; Höijer, J.; Bergman, P.; Björkhem-Bergman, L. Vitamin D supplementation to palliative cancer patients: Protocol of a double-blind, randomised controlled trial 'Palliative-D'. BMJ Supportive Palliat. Care **2017**, *7*, 458–463, doi:10.1136/bmjspcare-2017-001429.
8. Bergman, P.; Sperneder, S.; Höijer, J.; Bergqvist, J.; Björkhem-Bergman, L. Low vitamin d levels are associated with higher opioid dose in palliative cancer patients—Results from an observational study in sweden. PLoS ONE 2015, 10, e0128223, doi:10.1371/journal.pone.0128223.
9. Björkhem-Bergman, L.; Bergman, P. Vitamin D and patients with palliative cancer. BMJ Supportive Palliat. Care **2016**, *6*, 287–291, doi:10.1136/bmjspcare-2015-000921.
10. Helde-Frankling, M.; Bjorkhem-Bergman, L. Vitamin D in Pain Management. Int. J. Mol. Sci. **2017**, *18*, 2170, doi:10.3390/ijms18102170.
11. Bergman, P.; Norlin, A.C.; Hansen, S.; Rekha, R.S.; Agerberth, B.; Bjorkhem-Bergman, L.; Ekstrom, L.; Lindh, J.D.; Andersson, J. Vitamin D3 supplementation in patients with frequent respiratory tract infections: A randomised and double-blind intervention study. BMJ Open 2012, 2, doi:10.1136/bmjopen-2012-001663.
12. Norlin, A.C.; Hansen, S.; Wahren-Borgstrom, E.; Granert, C.; Bjorkhem-Bergman, L.; Bergman, P. Vitamin D3 Supplementation and Antibiotic Consumption—Results from a Prospective, Observational Study at an Immune-Deficiency Unit in Sweden. PLoS ONE 2016, 11, e0163451, doi:10.1371/journal.pone.0163451.

Supplementary Tables and Figures

Table S1. Baseline characteristics of patients who completed 12 weeks of intervention in the Palliative-D study (= per protocol population). *p*-values show comparison between the vitamin D group and the placebo group. Mann Whitney U was used for continuous variables and Fisher's exact test for categorical variables.

Variables	All (*n* = 150)	Vitamin D (*n* = 67)	Placebo (*n* = 83)	*p*-value
Age, median (IQR,) years	68 (61–75)	68 (61–76)	68 (61–75)	0.58
Male, No. (%)	74 (49)	34 (51)	40 (48)	0.89
Female, No. (%)	76 (51)	33 (49)	43 (52)	0.89
25-OHD, median (IQR), nmol/L	38 (30–45)	39 (30–46)	38 (30–45)	0.77
Fentanyl dose, median (IQR), ug/h	0 (0–12)	0 (0–25)	0 (0–12)	0.79
No. days on antibiotics, median (IQR), g/L	0 (0–2)	0 (0–0)	0 (0–3)	0.26
Albumin, median (IQR), g/L	32 (28–35)	32 (28–36)	32 (28–34)	0.80
Calcium, median (IQR), mmol/L	2.38 (2.31–2·44)	2.38 (2.29–2·43)	2.38 (2.33–2.46)	0.29
Creatinine, median (IQR), umol/L	72 (58–86)	72 (59–89)	72 (58–89)	0.64
CRP, median (IQR), mg/L	7 (2–22)	7 (1–24)	7 (2–18)	0.36
ESAS fatigue, median (IQR)	3 (1–5)	3 (1–5)	3 (1–5)	0.53
ESAS QoL, median (IQR)	4 (2–5)	3 (2–5)	4 (2–5)	0.25
EORTC QLQ-C15-PAL Q11 fatigue, median (IQR)	2 (2–3)	2 (2–3)	2 (2–3)	0.15
EORTC QLQ-C15-PAL Q15 QoL, median (IQR)	4 (3–5)	5 (3–5)	4 (3–5)	0.87
Type of cancer				
Brain	2	1	1	0.99
Breast	14	4	10	0.26
Upper gastrointestinal	35	16	19	0.85
Lower gastrointestinal	41	21	20	0.27
Gynecological	15	7	8	0.99
Head & Neck	0	0	0	0.99
Hematological	3	2	1	0.57
Lung	21	9	12	0.99
Melanoma	0	0	0	0.99
Prostate	14	7	7	0.99

Sarcoma	3	0	3	0.25
Urinary tract	3	0	3	0.25

In the placebo group one patient had two types of cancer (upper GI cancer and prostate cancer).S-25-OHD: S-25-hydroxyvitamin D, ESAS: Edmonton Symptom Assessment Scale (range 0–10), EORTC QLQ-C15-PAL: European Organization for Research and Treatment of Cancer Quality of Life Questionnaire C15 Palliative (fatigue range 1–4, QoL range 1–7), QoL: Quality of Life Q: Question.

Table S2. Beta coefficients for the adjusted variables at baseline in the longitudinal model for opioid dose (primary outcome) in the ITT-analysis. The opioid categories (fentanyl patches) listed were compared with "no opioids". The doses correspond to the available doses on the fentanyl patches except for 6 ug/h, which corresponds to 5 mg morphine per os. The oncological treatment categories were compared with "no treatment". 25-OHD = 25-hydroxyvitamin D.

**Baseline characteristics**	**Beta coefficient**	***p*-value**	**95% CI**
Male	1.52	0.60	−4.20 to 7.24
Age	0.11	0.44	−0.16 to 0.36
Colectomy–yes	−1.52	0.73	−7.17 to 10.22
25-OHD	−11.83	0.30	−34.16 to 10.49
**Opioid dose Fentanyl (ug/h)**			
6–12	15.14	<0.0001	7.0 to 23.3
25–37	42.7	<0.0001	33.0 to 52.4
50–67	62.9	<0.0001	54.5 to 71.3
100–175	127.7	<0.0001	111.5 to 143.8
250-	378.9	<0.0001	349.6 to 408.1
**Oncological treatment**			
Chemotherapy	−4.25	0.22	−11.1 to 2.6
Hormones	−8.31	0.09	−18.0 to 1.42
Target therapy	−5.79	0.33	−17.48 to 5.88

Table S3. Sensitivity analysis for the longitudinal linear mixed model, unadjusted ITT. Three different imputation models.

**Method**	**β / week**	**95% CI**	***p*-value**
Worst observation carried forward	−0.31	−0.74 to 0.11	0.15
Jump to reference multiple imputation	−0.39	−1.55 to 0.77	0.51
Copy increments in reference multiple imputation	−0.46	−1.43 to 0.51	0.36

β: beta coefficient; CI: confidence interval.

Table S4. Effect of vitamin D 4000 IU/day after 12 weeks (non-longitudinal analysis) on opioid dose, antibiotic use, fatigue and quality of life (QoL) compared to placebo in the per-protocol analysis, (n = 150). Adjustments were made for baseline value in both analysis and for age, sex, oncological treatment, baseline 25-OHD and colectomy in the adjusted analysis. Effect is presented as β-coefficient representing mean difference in change from baseline values between treatment arms after 12 weeks. The scale of EORTC-QLQ-C15 PAL Q15 has been reversed so negative value of beta is an improvement in QoL, in line with all the other outcomes where negative value is improvement * $p < 0.05$; ** $p < 0.01$.

**Variables**	**Unadjusted β (95% CI)**	***p*-value**	**Adjusted β (95% CI)**	***p*-value**
Fentanyl dose/hour	−7.00<br>−13.22 to −0.79	* 0.03	−6.1<br>−12.40 to 0.21	0.058
Days on antibiotics	−0.57<br>−2.21 to 1.08	0.50	−0.46<br>−2.14 to 1.22	0.59
ESAS fatigue	−1.12<br>−1.88 to −0.36	** 0.004	−1.11<br>−1.89 to −0.33	**** 0.006**
EORTC QLQ-C15 PAL Q11 fatigue	−0.23<br>−0.49 to 0.03	0.08	−0.25<br>−0.52 to 0.01	0.06
ESAS QoL	−0.58	0.13	−0.67	0.08

	−1.32 to 0.17		−1.42 to 0.09	
EORTC QLQ-C15 PAL Q15 QoL	−0.35<br>−0.79 to 0.09	0.12	−0.34<br>−0.79 to 0.11	0.13

CI: confidence interval, ESAS: Edmonton Symptom Assessment Scale (range 0–10), EORTC QLQ-C15 PAL: European Organization for Research and Treatment of Cancer Quality of Life Questionnaire C15 Palliative (fatigue range 1–4, QoL range 1–7), QoL: Quality of Life.

Table S5. Adverse events in the Palliative-D study in all randomised patients throughout the study period of 12 weeks. 244 patients were randomised to vitamin D 4000 IE/day or placebo: 206 patients finished 4 weeks, 169 finished 8 weeks and 150 patients finished 12 weeks; (vitamin D n = 67) and placebo (n = 83).

Adverse events	Vitamin D (n = 121)	Placebo (n = 123)
GI symptoms:<br>mild diarrhoea, nausea and/or stomach pain	2	1
Increased creatinine	1	0
Renal failure	0	1
Breathlessness	0	1
Hypercalcemia<br>(Albumin adjusted S-Ca >2.60)	2	2

Table S6. Opioid dose equivalent/conversion guide.

**Morphine, daily dose (mg)**		**Oxycodone, daily dose (mg)**		**Hydromorphone, daily dose (mg)**		**Fentanyl μg/h**
**po**	**iv/sc**	**po**	**iv/sc**	**po**	**iv/sc**	**transdermal**
20	7-10	10	7			12
40	15-20	20	15	4-8	2-4	12
60	20-30	30	20	8-12	4-6	25
80	30-40	40	30	12-16	6-8	25
100	35-50	50	35	14-20	7-10	37
120	45-60	60	45	18-24	9-12	50
160	60-80	80	60	24-32	12-16	50
220	80-110	110	80	32-44	16-22	75
320	120-160	160	120	48-64	24-32	100
400	150-200	200	150	60-80	30-40	125
500	185-250	250	185	74-100	37-50	150
580	215-290	290	215	86-116	43-58	175
680	255-340	340	255	102-136	51-68	200
760	285-380	380	285	114-152	57-76	225
860	320-430	430	320	128-172	64-86	250
940	350-470	470	350	140-188	70-94	275
1040	390-520	520	390	156-208	78-104	300

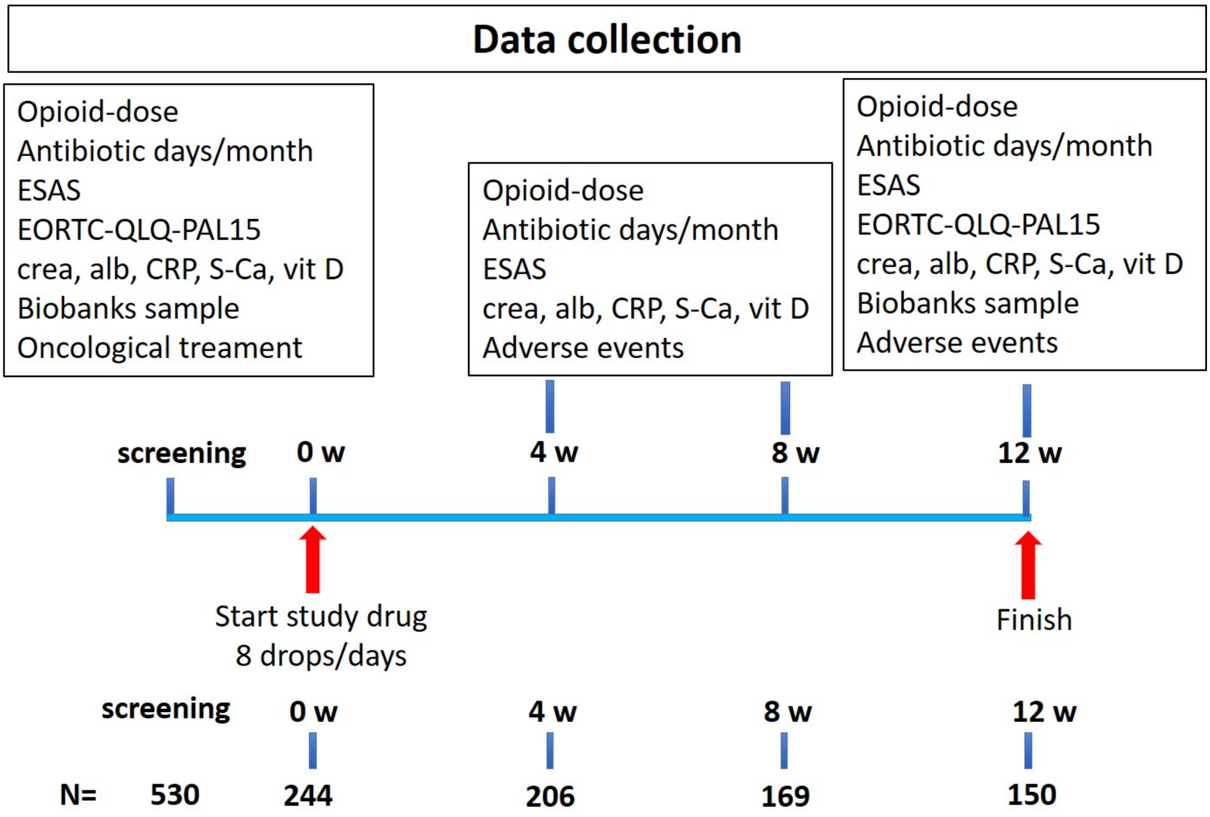

Figure S1. Schematic figure of data collection in the Palliative-D study.

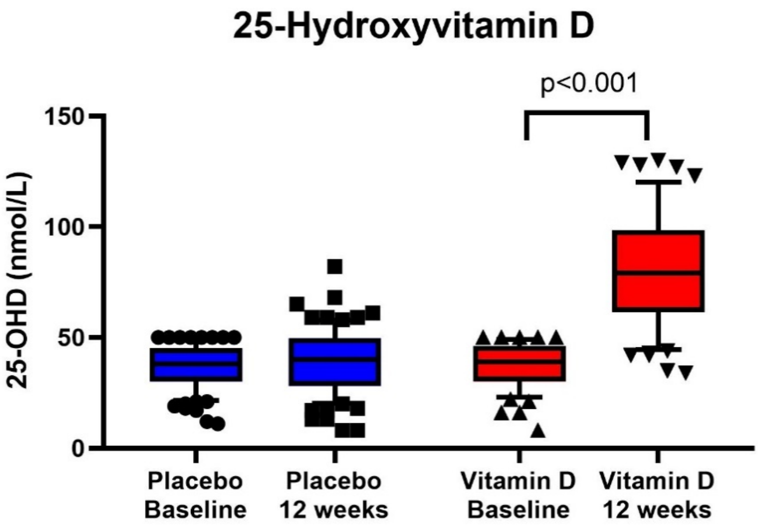

Figure S2. 25-hydroxyvitamin D levels (25-OHD) in plasma measured in the Palliative-D study at baseline and after 12 weeks of treatment with placebo (n = 83) or vitamin D 4000 IE/day (n = 67).

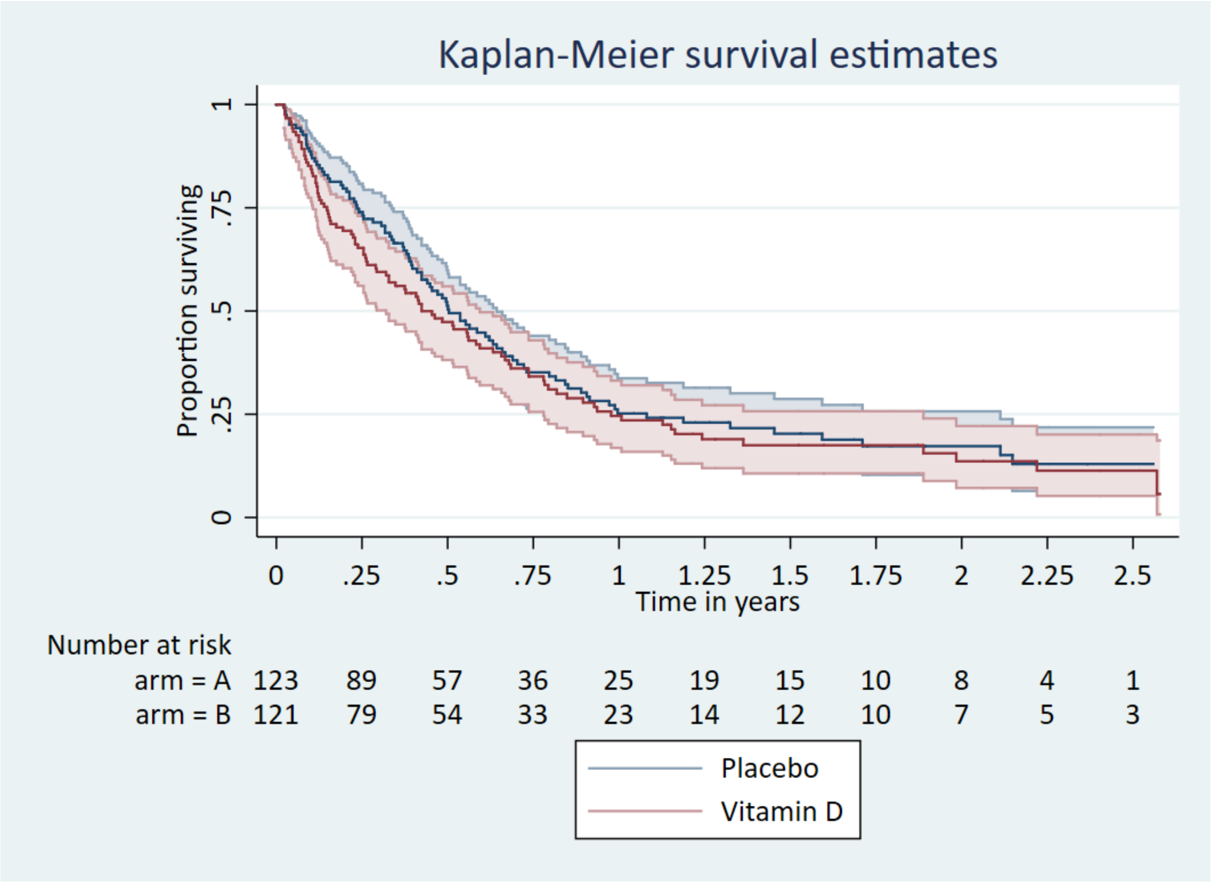

Figure S3. Kaplan-Meir plot of survival time in the Palliative D study throughout the study period (12 weeks) and follow-up for up to 2.5 years.

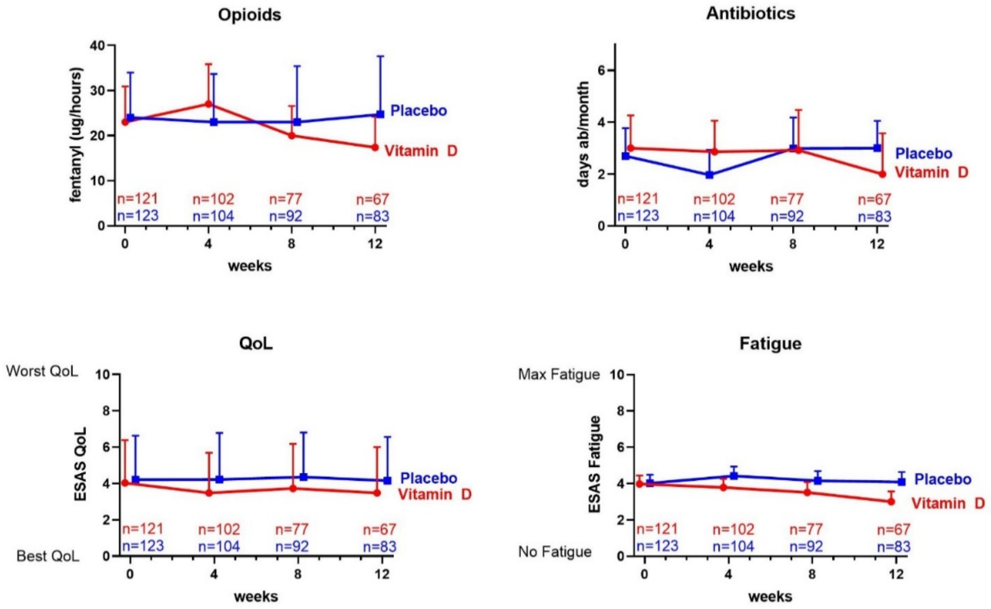

Figure S4. ITT-population: Raw-data. i.e. not adjusted for baseline. Change in opioid-doses, antibiotic consumption days of antibiotics/30 days, Quality of Life (QoL) and fatigue were assessed with ESAS in the Palliative-D study throughout the study period in all randomized patients (n = 244). The number of patients at each time point is presented. Points show mean of unadjusted raw data, + 95% CI.

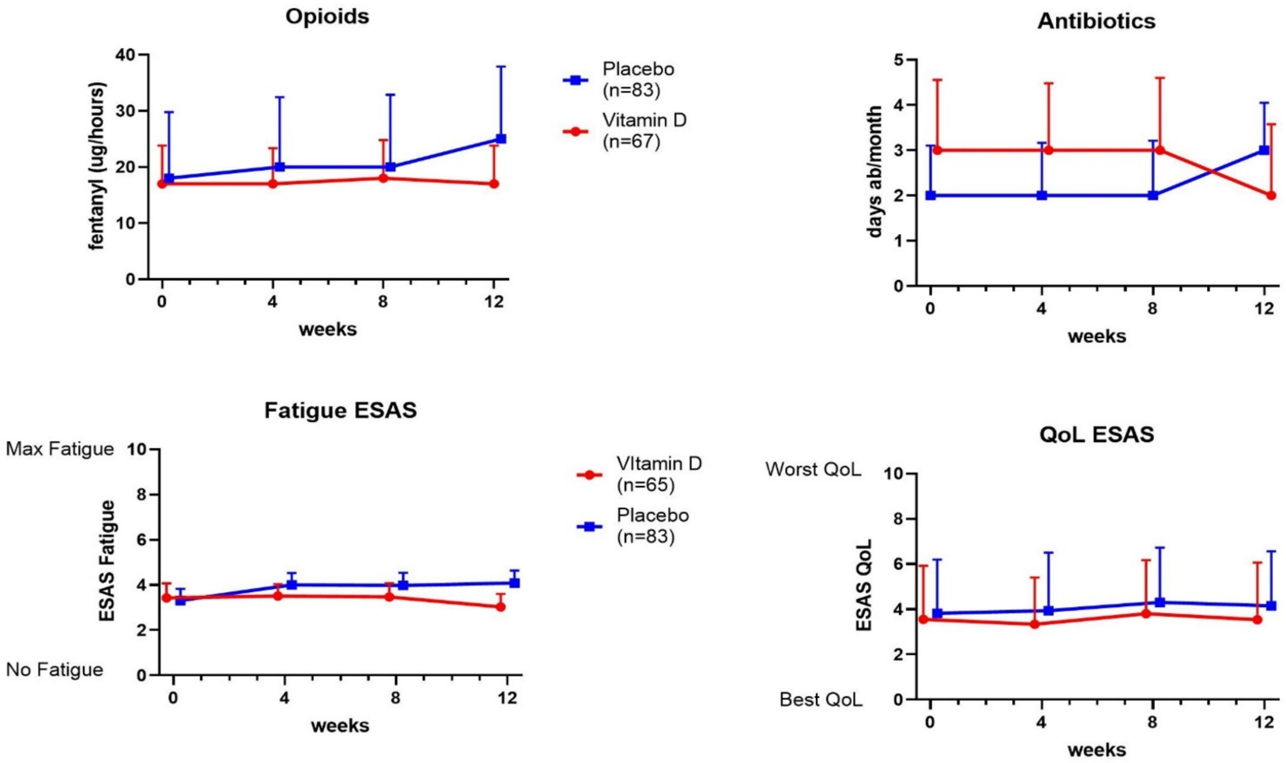

Figure S5. PP-population: Raw-data. i.e. not adjusted for baseline. Change in (A) opioid doses measured as fentanyl ug/hour throughout the study period (B) antibiotic use (days of antibiotics/30 days) (C) fatigue, and (D) QoL assessed with ESAS in the Palliative-D study in all patients completing the study. Points show mean of unadjusted raw data, +95% CI. The analysis is based on the 150 patients that completed the 12 weeks study period (vitamin D 4000 IE/day n = 67 and placebo n = 83), i.e. the per protocol study population.

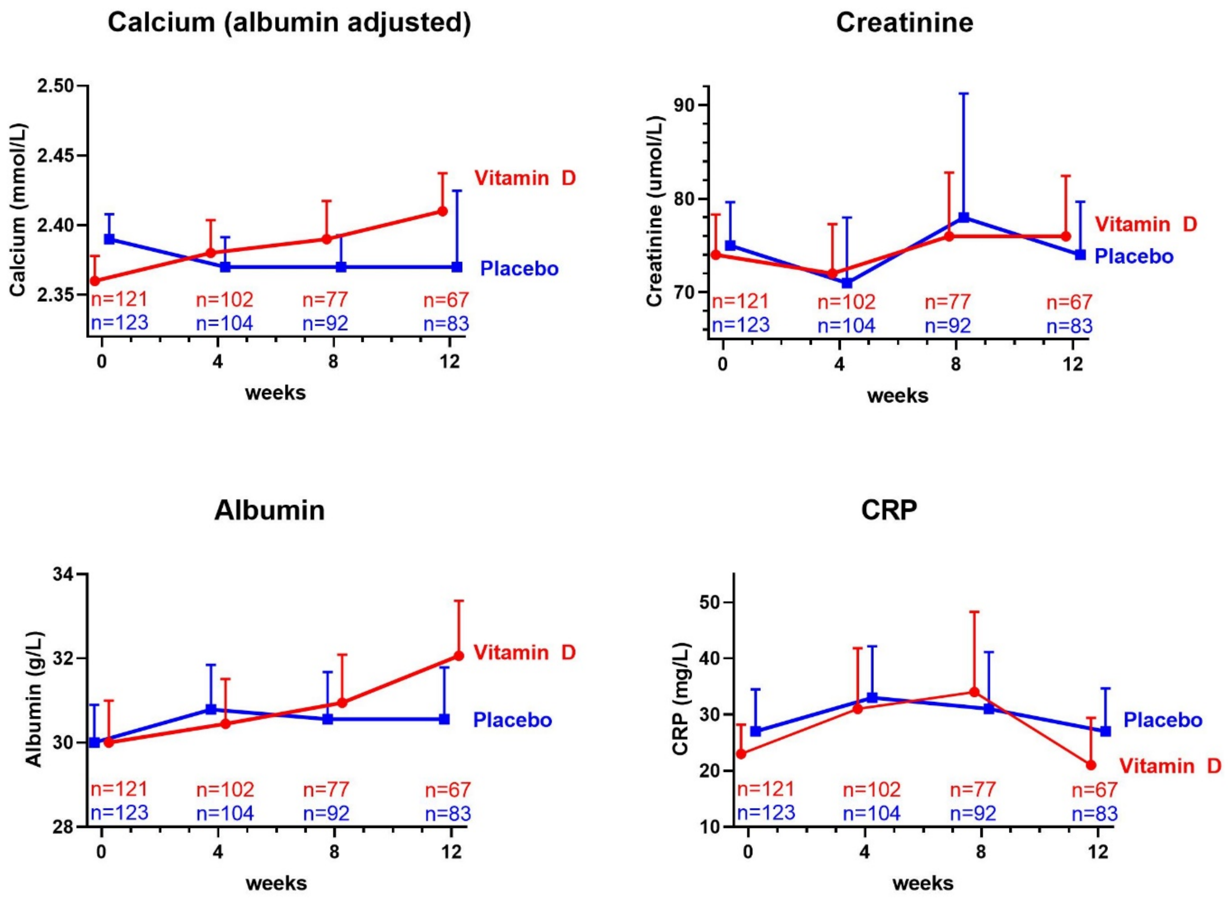

Figure S6. ITT-population: Raw-data. i.e. not adjusted for baseline. Levels of albumin adjusted calcium, C-reactive protein (CRP), albumin and creatinine in the Palliative-D study throughout the study period in all randomized patients ($n = 244$). Number of patients still participating in the study are indicated at each time point. Points show mean of unadjusted raw data, + 95% CI.

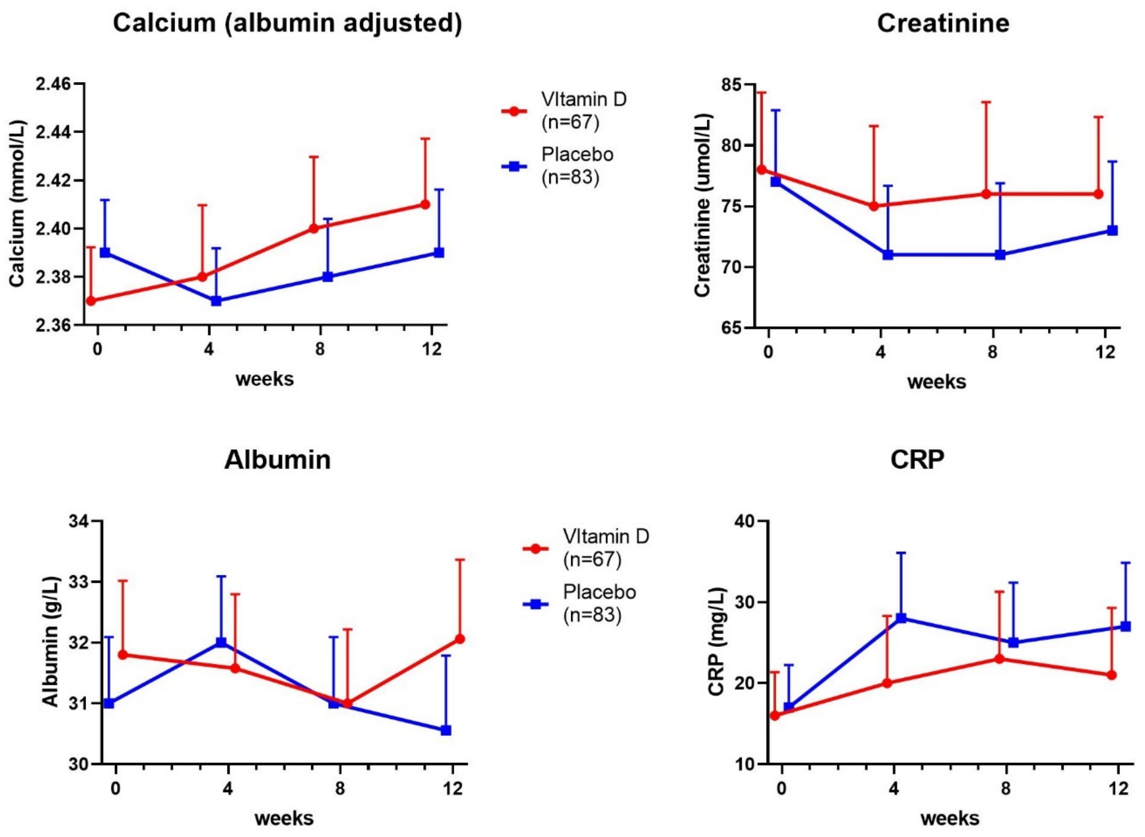

Figure S7. PP-population: Raw-data. i.e. not adjusted for baseline. Levels of albumin adjusted calcium, C-reactive protein (CRP), albumin and creatinine in all patients completing the Palliative-D study (*n* = 150). Points show mean of unadjusted raw data, +95% CI.
